# Genetic causes of neonatal and infantile hypercalcaemia

**DOI:** 10.1007/s00467-021-05082-z

**Published:** 2021-05-14

**Authors:** Caroline M. Gorvin

**Affiliations:** 1 Institute of Metabolism and Systems Research and Centre for Endocrinology, Diabetes and Metabolism, University of Birmingham, Birmingham, B15 2TT UK; 2 Centre of Membrane Proteins and Receptors (COMPARE), Universities of Birmingham and Nottingham, Birmingham, B15 2TT UK

**Keywords:** Calcium homeostasis, Genetic disease, Parathyroid hormone, Phosphate, Vitamin D

## Abstract

The causes of hypercalcaemia in the neonate and infant are varied, and often distinct from those in older children and adults. Hypercalcaemia presents clinically with a range of symptoms including failure to thrive, poor feeding, constipation, polyuria, irritability, lethargy, seizures and hypotonia. When hypercalcaemia is suspected, an accurate diagnosis will require an evaluation of potential causes (e.g. family history) and assessment for physical features (such as dysmorphology, or subcutaneous fat deposits), as well as biochemical measurements, including total and ionised serum calcium, serum phosphate, creatinine and albumin, intact parathyroid hormone (PTH), vitamin D metabolites and urinary calcium, phosphate and creatinine. The causes of neonatal hypercalcaemia can be classified into high or low PTH disorders. Disorders associated with high serum PTH include neonatal severe hyperparathyroidism, familial hypocalciuric hypercalcaemia and Jansen’s metaphyseal chondrodysplasia. Conditions associated with low serum PTH include idiopathic infantile hypercalcaemia, Williams-Beuren syndrome and inborn errors of metabolism, including hypophosphatasia. Maternal hypocalcaemia and dietary factors and several rare endocrine disorders can also influence neonatal serum calcium levels. This review will focus on the common causes of hypercalcaemia in neonates and young infants, considering maternal, dietary, and genetic causes of calcium dysregulation. The clinical presentation and treatment of patients with these disorders will be discussed.

## Introduction

Hypercalcaemia in the neonate and infant, although uncommon, can have serious long-term consequences, including nephrocalcinosis that may cause permanent kidney damage, osteoporosis and neurodevelopmental impairments, and has varied etiologies (Table 1). Hypercalcaemia is defined as a serum calcium concentration two standard deviations greater than the normal mean. An accurate determination of the serum calcium concentration with age-appropriate normal ranges (Table 2) is required as serum calcium levels are higher in newborns and preterm infants and decline with age [1, 3]. In late gestation, calcium is actively transported across the placenta to facilitate mineralisation of the foetal skeleton, resulting in relative foetal hypercalcaemia. At birth, a transient decrease in serum calcium occurs as active placental transport ends upon cutting the umbilical cord [4]. This transient hypocalcaemia is more pronounced in preterm infants, due to the early discontinuation of transplacental calcium transport and reduced responses to parathyroid hormone (PTH) and infants of diabetic mothers, caused by neonatal functional hypoparathyroidism due to maternal hypomagnesemia [5]. Hypercalcaemia presents clinically with a range of symptoms including failure to thrive, poor feeding, constipation, polyuria, irritability, lethargy, seizures and hypotonia. When hypercalcaemia is suspected, an accurate diagnosis will require an evaluation of potential causes (e.g. family history or diet) and assessment for physical features (such as dysmorphology, granulomatous disease or subcutaneous fat deposits), as well as biochemical measurements. These measurements should include total and ionised serum calcium, serum albumin to calculate corrected serum calcium, serum phosphate, creatinine, intact PTH, vitamin D metabolites and urinary calcium, phosphate and creatinine [6].

**Table 1:** Causes of neonatal/infantile hypercalcaemia

**Disease**		**Causes**
**High serum PTH**	Neonatal severe hyperparathyroidism (NSHPT)	Inactivating mutations in the CaSR gene – most often homozygous or compound heterozygous
	Familial hypocalciuric hypercalcaemia (FHH)	Inactivating mutations in the CaSR gene – most often heterozygous; Heterozygous inactivating mutations in Gα11 and AP2σ genes (encoded by *GNA11* and *AP2S1*, respectively)
	Secondary hyperparathyroidism/transient hypercalcaemia	Maternal hypoparathyroidism or maternal hypocalcaemia
**Low serum PTH**	Dietary causes	Excess calcium in enriched formula; Low phosphate by parenteral feeding, incorrectly prepared formulas; Excess vitamin D intake by the infant or the breast-feeding mother; Excess vitamin A intake by enteral feeding
	Jansen’s metaphyseal chondrodysplasia	Inactivating mutations in the *PTH1R* gene
	Idiopathic infantile hypercalcaemia (IIH)	Homozygous inactivating mutations in the cytochrome P450, 24-hydroxylase gene (*CYP24A1*); Homozygous inactivating mutations in the sodium-phosphate co-transporter NaPi-IIa (encoded by *SLC34A1*)
	Williams-Beuren syndrome	Microdeletion of 26–28 genes on 7q11.23
*Inborn errors of metabolism*	Hypophosphatasia	Inactivating mutations in the tissue non-specific isoenzyme of alkaline phosphatase (TNSALP, encoded by *ALPL*)
	Blue-diaper syndrome	A mutation in the pro-protein convertase subtilisin/kexin type 1 (*PCSK1*) has been reported in one case, although this infant did not have hypercalcaemia. Genetic cause unknown.
	Congenital glucose-galactose malabsorption	Homozygous inactivating mutations in the sodium-dependent glucose transporter-1 (SGLT-1)
	Congenital lactase deficiency	Homozygous or compound heterozygous mutations in the lactase (LCT) gene
Miscellaneous	Subcutaneous fat necrosis	Necrosis and granulomatous infiltrate following traumatic birth or therapeutic hypothermia
	Bartter’s syndrome	Cases identified in infants with mutations of the sodium-potassium-chloride co-transporter NKCC2 (encoded by *SLC12A1*) and the potassium channel ROMK (encoded by *KCNJ1*)
	Intrauterine growth retardation, metaphyseal dysplasia, adrenal hypoplasia congenita, and genital anomalies (IMAGe syndrome)	Heterozygous mutations in the cyclin-dependent kinase inhibitor 1C (*CDKN1C*) gene
	Hyperthyroidism	Maternal Graves’ disease Neonatal hypothyroidism e.g. autosomal dominant non-autoimmune hyperthyroidism, McCune-Albright syndrome
	Adrenal insufficiency	Reduced glomerular filtration rate and/or increased 1α-hydroxylase activity
	PTHrP-secreting tumours	PTHrP binding to PTH1R
	Drugs	Thiazides and lithium

**Table 2:** Age-specific reference intervals of serum calcium concentrations

Age range	Total serum calcium		Ionised serum calcium	
	(mg/dL)	(mmol/L)	(mg/dL)	(mmol/L)
Cord blood	8.2–11.2	2.05–2.80	5.20–6.40	1.30–1.60
Neonate (24 h)	–	–	4.40–5.44	1.10–1.36
Neonate (5 days)	–	–	4.88–5.92	1.22–1.38
Birth to 90 days	8.0–11.3	2.0–2.8	–	–
91–180 days	8.9–11.2	2.2–2.8	–	–
181–364 days	9.0–11.3	2.3–2.8	–	–
1–3 years	8.9–11.1	2.2–2.8	4.80–5.52	1.20–1.38

Information on total serum calcium from Roizen et al. [1]. These individuals were reported to have a plasma albumin in the normal range. Cord blood calcium and ionised serum calcium concentrations were adapted from Stokes et al. [2]

“–” inserted where information was not available

**Fig. 1:**
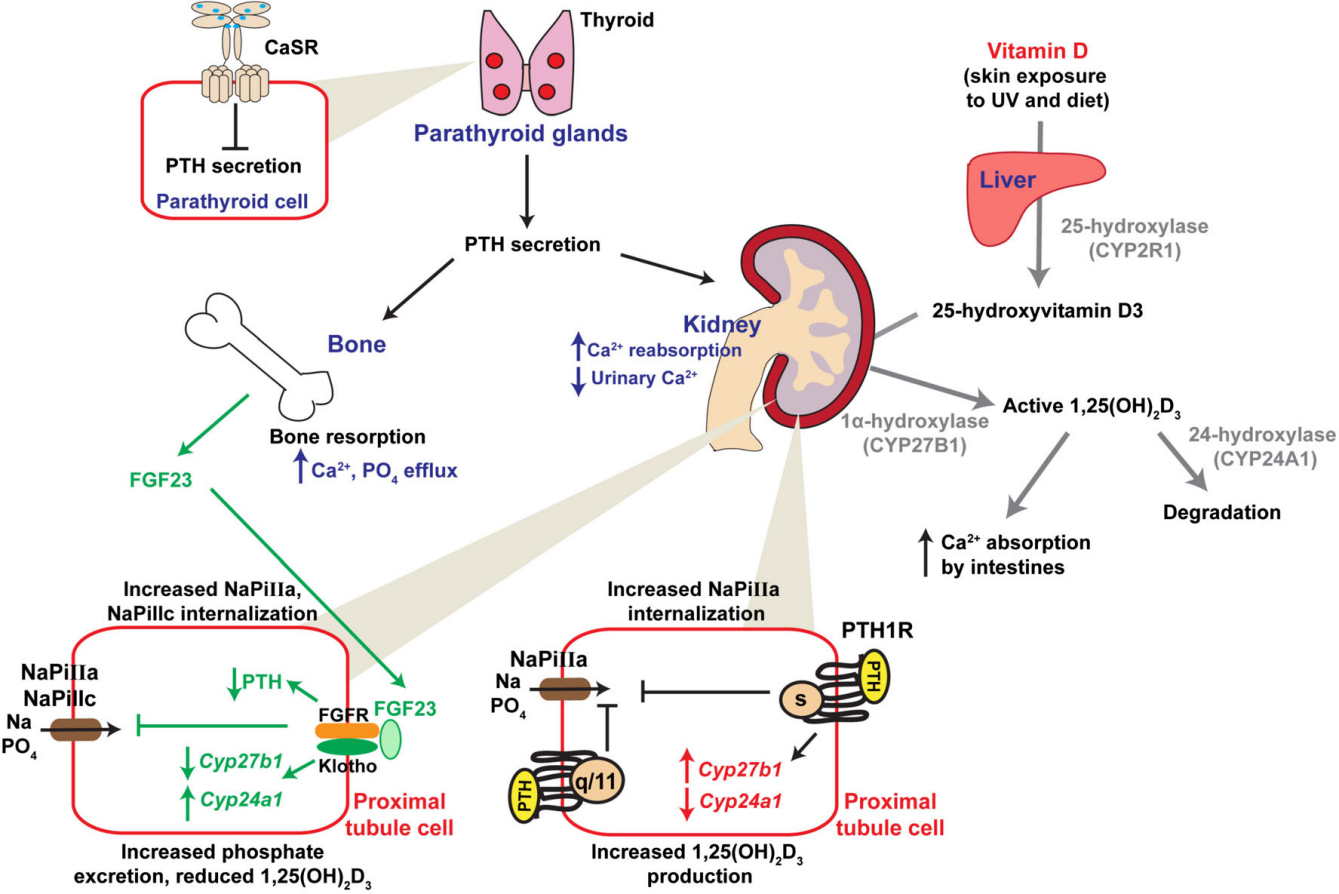
Calcium regulation at the parathyroid-bone-kidney-gut axis. Serum calcium concentrations are detected by the calcium-sensing receptor (CaSR) on parathyroid cell surfaces. CaSR signalling inhibits PTH secretion. In the presence of low serum calcium or magnesium, this inhibition is relieved, allowing PTH secretion to occur. PTH acts at bone to increase resorption and calcium release, and kidney reducing calcium excretion. At kidney proximal tubules, PTH1R activates signalling pathways that stimulate NaPi-IIa internalisation which reduces phosphate uptake and alters *CYP27B1* and *CYP24A1* expression, resulting in 1,25(OH)_2_D_3_ synthesis. Vitamin D activation involves hydroxylation in the liver by 25-hydroxylase (CYP2R1) to form 25-hydroxyvitamin D3, and at the kidney, by 1α-hydroxylase (CYP27B1), to generate the active 1,25(OH)_2_D_3_, which can bind to the vitamin D receptor. Inactivation requires 24-hydroxylase (CYP24A1). In the FGF23-klotho axis (shown in green), FGF23 is secreted by osteocytes and binds to the FGF receptor (FGFR)-klotho complex at kidney proximal tubules, where it reduces PTH transcription and plasma membrane expression of NaPi-IIa and NaPi-IIc, resulting in increased phosphate excretion. FGF23 also reduces synthesis of 1,25(OH)_2_D by inhibiting *CYP27B1* and stimulating *CYP24A1*.

Serum calcium levels are maintained by interplay between the parathyroid glands, kidney, gut and bone (Fig. 1). When serum calcium concentrations are low, the parathyroid glands synthesise and secrete PTH, which acts on PTH receptors (PTH1R) on bone and kidney cells. At bone, this leads to enhanced resorption and consequent increased efflux of calcium and phosphate. At the kidney, PTH reduces calcium excretion and enhances 1,25(OH)_2_D_3_ synthesis, which stimulates intestinal calcium absorption. The net effect is a normalisation of serum calcium levels [3, 7–9]. Elevations in serum calcium outside the normal range activate the calcium-sensing receptor, a G protein-coupled receptor (GPCR) expressed on parathyroid cell surfaces, which suppresses PTH secretion [3, 7–9]. Vitamin D activation, and subsequent inactivation, requires a series of enzymatic reactions beginning with hydroxylation in the liver by 25-hydroxylase (CYP2R1) to form 25-hydroxyvitamin D3. A second hydroxylation in the kidney, by 1α-hydroxylase (CYP27B1), generates the active form 1,25(OH)_2_D_3_, which can bind to the vitamin D receptor. Inactivation requires 24-hydroxylase (CYP24A1) [3, 7–9]. FGF23 secretion by osteocytes is stimulated by 1,25(OH)_2_D_3_. At the kidney proximal tubule, FGF binding to the FGF receptor (FGFR)–klotho complex stimulates signalling pathways that decrease PTH transcription and reduce plasma membrane expression of sodium–phosphate transporters resulting in reduced phosphate uptake and increased urinary excretion. FGF23 also reduces synthesis of 1,25(OH)2D by inhibiting *CYP27B1* and stimulating *CYP24A1* [3, 7] (Fig. 1). In the developing foetus, calcium homeostasis is regulated, in part, by the parathyroid hormone-related peptide (PTHrP), while PTH, 1,25(OH)_2_D_3_ and FGF23 are low in foetal circulation [10].

## Causes of neonatal hypercalcaemia

The causes of hypercalcaemia in the neonate are often distinct from those in older children and adults. This review will focus primarily on genetic causes of hypercalcaemia in neonates and young infants. Neonatal hypercalcaemia can be due to maternal or neonatal factors and can be divided into high or low PTH disorders (Table 1).

## High serum PTH

### Neonatal severe hyperparathyroidism

Neonatal severe hyperparathyroidism (NSHPT) (OMIM #239200) is a rare condition in which affected infants present with marked hypercalcaemia, skeletal demineralisation and failure to thrive, and can be fatal if untreated [11]. In 1993, inactivating mutations in the gene encoding the calcium-sensing receptor (CaSR) (Fig. 1) were identified to cause NSHPT, and familial hypocalciuric hypercalcaemia (FHH), a milder hypercalcaemic disorder [11]. In the majority of cases (~ 85%), NSHPT is caused by homozygous or compound heterozygous mutations, while heterozygous mutations cause FHH [12]. However, NSHPT has also been described in a small number of patients with heterozygous CaSR mutations, and it has been hypothesised that the clinical presentation of patients with heterozygous mutations is likely influenced by the location of the mutant residue, as well as intrauterine exposure to maternal calcium and vitamin D, and the presence of other CaSR polymorphisms that could act as genetic modifiers to influence activity of the receptor [12–14].

Individuals with NSHPT usually present within the first 2 weeks post-partum with serum calcium levels of > 4.5 mmol/L [12]. Serum calcium values are higher in patients with homozygous or compound heterozygous mutations and in individuals with nonsense or truncation mutations that severely impact receptor structure [12]. PTH values are high in > 80% of NSHPT patients [12]. The most commonly reported symptoms in NSHPT are skeletal under-mineralisation and/or osteopenia, failure to thrive and hypotonia; while respiratory distress and lethargy are observed in a third of individuals, and dehydration, constipation and nausea/vomiting are observed in < 20% of NSHPT patients [12].

Parathyroidectomy is the preferred treatment for NSHPT, with bisphosphonates used to reduce hypercalcaemia prior to surgery [15]. Surgery should be performed early to avoid neuromotor restrictions, which may persist after otherwise successful surgery, and has been correlated with microcephaly and the duration or amplitude of hypercalcaemia [16]. The CaSR-positive allosteric modulator, cinacalcet, which enhances receptor sensitivity to extracellular calcium, has successfully reduced PTH and serum calcium in some NSHPT patients [15, 17]. The efficacy of cinacalcet is genotype dependent and may be ineffective in some patients [15, 17]. However, cinacalcet is likely to be increasingly utilised in NSHPT, due to its rapid effect on PTH levels and serum calcium [12].

### Familial hypocalciuric hypercalcaemia type-1

FHH (familial hypocalciuric hypercalcaemia type-1 (FHH1), OMIM #145980) is an autosomal dominant disorder characterised by lifelong elevated serum calcium levels, high (approximately one-third) or inappropriately normal PTH concentrations and low kidney calcium excretion [12, 18, 19]. FHH1 is usually benign, and although its biochemical features have considerable overlap with typical primary hyperparathyroidism (PHPT), hypercalcaemia in FHH remains persistent following parathyroidectomy. It is therefore important to distinguish between typical PHPT and FHH to avoid unnecessary surgery [12, 18, 19]. This should involve a combination of genetic analysis, measurement of urinary calcium/creatinine ratio (UCCR) (cut-off of < 0.02 for FHH1) and examination of other symptoms, including kidney stones, fractures and cardiovascular events that are more common in PHPT [12, 18, 19].

FHH is rarely detected in infants, and the majority of patients with FHH are asymptomatic (~ 71%). However, some individuals have hypercalcaemic symptoms including constipation, fatigue, headaches, muscle cramps, nausea and vomiting, while other patients present with associated features including nephrocalcinosis/nephrolithiasis, osteoporosis and/or fractures and pancreatitis [12]. Treatment of symptomatic hypercalcaemia with cinacalcet has been reported in some adults and young children with FHH1 [20, 21]. However, the safety and efficacy have not been thoroughly examined in children, and the FDA has not approved its use in this age group [21].

In the last decade, the genetic heterogeneity of FHH has emerged, with mutations in the Gα11 protein [22], by which CaSR signals, and the adaptor protein-2 sigma subunit (AP2σ) [23], which facilitates CaSR internalisation, demonstrated to cause FHH. AP2σ mutations have been reported to cause a more severe FHH phenotype than those associated with CaSR mutations, with patients often exhibiting higher serum calcium values, symptomatic hypercalcaemia and additional features including recurrent pancreatitis [24]. Despite this, AP2σ mutations were not identified in a cohort of NSHPT patients [25], which may be because homozygous mutations in AP2σ are associated with lethality in mouse models [26], and therefore may not be tolerated in humans. Cinacalcet has been shown to normalise serum calcium and PTH and resolve hypercalcaemic symptoms (headaches, abdominal pain, vomiting, fatigue, musculoskeletal pain) in patients with *AP2S1* and *GNA11* mutations [27, 28].

### Maternal hypocalcaemia

Foetal serum calcium levels are maintained at a higher concentration than maternal calcium concentrations to allow skeletal formation and mineralisation to take place [29]. Maternal hypoparathyroidism or maternal hypocalcaemia may cause secondary hyperparathyroidism in the neonate [30]. In the presence of severe maternal hypocalcaemia, foetal hypocalcaemia will develop, resulting in stimulation of the foetal parathyroid glands and development of hyperparathyroidism [31]. In severe cases, foetal skeletal demineralisation can lead to sub-periosteal bone resorption, osteitis fibrosa cystica, bowing of the long bones, fractures, low birth weight and foetal death [31]. In most cases, measuring maternal serum calcium and determining whether a familial or maternal history of hypercalcaemia exists are sufficient for diagnosis. The hypercalcaemia in the neonate is usually resolved within a few weeks [31], although some infants have been treated with vitamin D [32].

## Low serum PTH

### Nutritional causes

Hypercalcaemia can occur in the neonate or young infant following ingestion of excess calcium in enriched formulas. Low dietary phosphate can also cause hypercalcaemia, due to suppression of FGF23 or elevations in 1,25(OH)_2_D_3_. This is rare since the introduction of breast milk fortifiers, which contain 30–40 mg kg^−1^ day^−1^ of phosphate [3]. Phosphate depletion occurs more frequently in very low birth weight pre-term infants given inappropriately supplemented parenteral nutrition often leading to refeeding syndrome, characterised by hypokalemia, hypoglycaemia and hypophosphatemia [33]. In particular, hypophosphatemia has been associated with parenteral nutrition with a high amino acid content, and formulas with equimolar (0.75:1 to 1:1) Ca^2+^:PO_4_ ratios, along with careful monitoring of electrolytes including phosphorus have been recommended to prevent hypercalcaemia and hypophosphatemia [33, 34]. Hypophosphatemia has been described in some infants given correctly prepared formulas, and it has been suggested that bioavailability of phosphorus may be suboptimal in some infants in the context of gastrointestinal disease or tube feeding [35]. Formula change and/or phosphate supplementation improved hypophosphatemia in these infants [35].

Excessive vitamin D intake, either by the infant or the breast-feeding mother can lead to increased intestinal calcium and phosphate absorption, enhanced bone resorption, and consequently hypercalcaemia and hyperphosphatemia [6]. Rehydration therapy with furosemide usually resolves hypercalcaemia in these infants, although bisphosphonates have been used in patients in which 25(OH)D remained high [36].

Hypercalcaemia has been reported in some infants receiving enteral feeding formulas containing excess vitamin A. The metabolite of vitamin A, retinoic acid, can bind to receptors on osteoclasts and osteoblasts, resulting in increased bone resorption and decreased bone formation [6].

### Jansen’s metaphyseal chondrodysplasia

Jansen’s metaphyseal chondrodysplasia (JMC, OMIM #156400) is an autosomal dominant disease characterised by short-limbed short stature, deformed, under-mineralised bones, chronic hypercalcaemia and hyperphosphaturia. Most patients have low or undetectable serum PTH levels and elevated serum markers of bone turnover [37].

JMC is very rare, with fewer than 30 reported cases to date [38]. It is caused by mutations in the receptor for PTH and PTHrP, parathyroid hormone 1 receptor (PTH1R) [39], a GPCR that is expressed in cells of bone and kidney. The majority of mutations arise *de novo*, although some familial cases have been described [40]. All reported mutations occur in three residue positions His223, Thr410 and Ile458 (located in transmembrane helices 2, 6 and 7, respectively), which are located in a bundle at the cytoplasmic face of the receptor [39, 41]. Structural models indicate these residues contribute to a network that controls the outward movements of the transmembrane helices, allowing formation of a cavity at which G proteins can engage with the cytoplasmic region of the receptor during activation [37]. Such a critical role for these residues in receptor activation is consistent with in vitro findings that JMC mutations are associated with ligand-independent constitutive activation of cAMP signalling [39]. This is despite reduced receptor cell surface expression.

JMC initially presents as a generalised skeletal dysplasia, but as patients age it becomes apparent that the metaphyses are preferentially affected, with progressive expansion and irregular ossification, resulting in short and bowed legs and short hands with clubbed fingers [38, 40]. This can be explained by the very high expression of PTH1R in proliferating chondrocytes of the growth plates. Patients with JMC may also exhibit micrognathia, hypertelorism, high-arched palate, delayed tooth eruption or impaction and a sclerotic skull base [38]. Such growth abnormalities are often not apparent at birth and initial symptoms may present as difficulty in feeding, vomiting, dehydration or respiratory distress within the first few months of life [38, 42]. However, X-rays of infants with JMC show bony lesions comprising rachitic changes, radiolucency and irregularities of the metaphyses of the long bones [30].

The metaphyseal irregularities and skeletal hypo-mineralisation can be mistaken for hypophosphatemic rickets; however, the biochemical findings are inconsistent with this diagnosis [43]. Neonates with JMC have severe, but largely asymptomatic hypercalcaemia, hypophosphatemia, hyperphosphaturia, elevated circulating levels of 1,25-dihydroxyvitamin D_3_, elevated serum alkaline phosphatase, and low or undetectable levels of PTH and PTH-related peptide (PTHrP) [44]. Serum calcium remains high throughout life but reduces with age [44]; however, PTH levels remain suppressed at or below the lower limit of the reference range [45].

Attempts to perform genotype-phenotype correlations have been made, though there are inconsistencies in their findings. Early studies indicated that patients with His223 mutations may have more severe hypercalcaemia. However, it is now recognised that there is considerable variability even within families [40, 44]. There is some evidence that the constitutive activity of the Thr410Arg mutant PTH1R is not as marked as for other receptor mutations, and clinical features in the affected family are less severe, including milder bone dysplasia and serum calcium and phosphate levels occasionally within the normal range [43]. However, it is difficult to confirm these correlations due to the paucity of JMC cases.

There are currently no effective treatments for JMC, although the use of bisphosphonates in older children to reduce hypercalcaemia has been reported [42, 46]. Recently, the effectiveness of an inverse agonist of the PTH1R was tested in a transgenic mouse model expressing the PTH1R-His223Arg mutation in osteoblast cells [37]. Mutant mice showed significant improvements in skeletal parameters including excess trabecular bone mass, bone marrow fibrosis and levels of bone turnover markers, although there was no effect on bone length [37]. Further studies of such PTH1R ligand analogues could reveal new treatment options for JMC patients.

### Idiopathic infantile hypercalcaemia

Between the 1930s and 1950s, there was an increase in reported cases of hypercalcaemia in children by clinicians in the UK. Initial observations included failure to thrive, hypercalcaemia and distinct facial features (described as “elfin”). While the hypercalcaemia was transient in some, the facial abnormalities, developmental delay and mental retardation persisted [47]. In 1953, Lightwood and Stapleton suggested that a distinction should be made between those individuals with “syndromic” hypercalcaemia accompanied by distinct facial features and mental retardation, and those with classical features of hypercalcaemia, including thirst, dehydration and polyuria [47]. This latter milder form was termed idiopathic infantile hypercalcaemia (IIH, OMIM #143880), and the former named Williams-Beuren Syndrome [8] (see next section). The increased prevalence of IIH in the 1950s in the UK was linked to the high dose of vitamin D fortification in milk and cereals, an initiative that had begun to prevent rickets in the population [47]. An oversensitivity to vitamin D was suggested to be the cause of hypercalcaemia in those individuals with IIH [47].

IIH classically presents in the first year of life with typical symptoms of hypercalcaemia, including failure to thrive, weight loss, dehydration, polyuria, vomiting and lethargy [48]. Hypercalcaemia results from intestinal hyperabsorption [49] and is accompanied by suppressed intact PTH and hypercalciuria [8]. Nephrocalcinosis is also observed on kidney ultrasonography in infants from a few months of age [8]. Hypercalcaemia generally resolves before the age of three years, although nephrocalcinosis and persistent hypercalciuria can continue to be common [50]. If diagnosed early, the prognosis of IIH patients can be improved, and extent of nephrocalcinosis limited [50]. Individuals with IIH may have elevated levels of serum 1,25(OH)_2_D_3_, although some have inappropriately normal levels [48].

Although early studies had established an association with vitamin D supplementation, an explanation for this link was only provided in 2011 when researchers identified recessive mutations in the *CYP24A1* gene in several individuals with IIH [8]. *CYP24A1* encodes the cytochrome P450 enzyme 24-hydroxylase, which inactivates 1,25(OH)_2_D_3_ (Fig. 1) [48]. In this initial report, *CYP24A1* mutations were nonsense or missense and inherited in the homozygous or compound heterozygous state [8]. Subsequently, further mutations were identified that were always bi-allelic, although there is some evidence that heterozygous carriers have high normal serum calcium, increased 1,25(OH)_2_D_,_ and low levels of PTH [48, 51]. Functional analysis revealed the mutations identified in IIH patients led to complete loss of *CYP24A1* enzymatic activity [8]. Some *CYP24A1* inactivating mutations have been identified in adults with recurrent kidney stone disease and chronic kidney failure [52, 53], with only a subset known to have hypercalcaemia in childhood. Moreover, some *CYP24A1* mutations, which tend to be associated with milder hypercalcaemia, are present at a high frequency in the Caucasian population (4–20% on dbSNP), indicating that IIH may go undetected if not challenged by vitamin D supplementation, resulting in increased lifetime risk of nephrocalcinosis and nephrolithiasis [54].

In 2016, mutations in a second gene, *SLC34A1*, which encodes the kidney proximal tubule sodium-phosphate co-transporter NaPi-IIa, were identified in patients with IIH [9]. Again, these mutations were autosomal recessive and led to inactivation of the NaPi-IIa protein [9]. This subset of patients not only have the classical symptoms of hypercalcaemia and suppressed PTH but also exhibit hypophosphatemia due to kidney phosphate wasting [8]. Functional expression of the IIH-associated mutant NaPi-IIa demonstrates that the transporter fails to traffic normally to the surface of cells due to intracellular retention, resulting in defective kidney proximal tubular phosphate reabsorption and hypophosphatemia [8]. Subsequently, the low levels of serum phosphate and FGF23 induce increases in *CYP27B1* expression and 1a-hydroxylase activity; while inhibiting *CYP24A1* expression and 24-hydroxylase activity, which have the combined effect of increasing 1,25-(OH)_2_D_3_ and hypercalcaemia [8]. Mutations in *SLC34A1* have also been described in a consanguineous family with an affected individual presenting with renal Fanconi’s syndrome, hypophosphatemic rickets, hypercalciuria and elevated 1,25(OH)_2_D_3_ levels [55], and in heterozygous adults with hypophosphatemia and urolithiasis [56]. The clinical biochemistry during infancy in these individuals was not reported, although the younger sister of the individual with Fanconi’s syndrome and hypophosphatemic rickets had hypercalcaemia, suppressed PTH and elevated 1,25(OH)_2+_D_3_ levels [55]. Thus, although hypercalcaemia may resolve within the first few years in most individuals, patients may develop other sequelae including nephrolithiasis later in life.

### Williams-Beuren syndrome

Williams-Beuren syndrome, also known as Williams syndrome (OMIM #194050), is a rare genetic multisystem disorder that affects the cardiovascular, connective tissue and central nervous systems. It affects ~1 in 7500–10,000 live births [57, 58]. Although symptoms vary between cases, distinct (“elfin-like”) facial features including microcephaly, broad forehead, rounded cheeks, epicanthal folds, depressed nasal bridge, small mandible and prominent ears are usually present [59, 60]. Dental abnormalities including microdontia, hypoplastic enamel and upper and lower teeth that do not meet properly (malocclusion) can occur in up to 90% of cases and stellate patterns in the iris are common [60]. Many of the developmental defects including mental retardation and poor motor development observed in Williams syndrome are not detected until later in childhood. However, musculoskeletal abnormalities including depression of the breast bone (pectus excavatum), scoliosis or kyphosis are apparent earlier in childhood [60]. In neonates, low birth weight, respiratory distress, feeding difficulties, recurrent vomiting, colic and diarrhoea are common, and two-thirds of infants are small for gestational age [30, 60]. Short stature remains a feature in individuals with Williams syndrome [60]. Soft tissue abnormalities including hernias and skin hyperelasticity, as well as hypotonia and chronic urinary tract infections are often observed in Williams syndrome [60]. Cardiovascular defects are the most common cause of death in Williams syndrome patients and are present in over 90% of affected neonates [58, 59]. Cardiac defects include pulmonary stenosis and supraventricular aortic stenosis, and patients have symptoms of fatigue, chest pains, dizziness and heart murmurs [59].

There are discrepancies in the reported frequency of hypercalcaemia in association with Williams syndrome, ranging from as low as 5% to up to 50% [60, 61]. The high serum calcium levels are usually resolved within the first few years but may recur during puberty [3]. Circulating calcitriol levels are elevated in some patients, and PTH levels are low [3]. Hypercalcaemia is frequently symptomatic with many infants presenting with dehydration, fatigue, abdominal pain and irritability [3, 61]. There is no consensus regarding the pathogenesis of hypercalcaemia, although decreased calcitonin production and abnormal vitamin D metabolism have been implicated [3, 30].

Williams-Beuren syndrome is a microdeletion disorder caused by the loss of 26–28 genes on 7q11.23 [61, 62]. Most cases occur spontaneously, although familial cases have been reported in which there is autosomal dominant inheritance [60]. Hemizygosity of the elastin gene (*ELN*) is seen in over 90% of cases and has been demonstrated to be responsible for the vascular and connective tissue defects in Williams syndrome [62, 63]. Deletion of the LIM kinase gene *LIMK1* has been reported to contribute to the visuo-spatial cognition problems associated with Williams syndrome [64]. Deletion of the other 24-26 genes on 7q11.23 is likely to be responsible for other symptoms observed in Williams syndrome patients, including hypercalcaemia. One candidate, the Williams syndrome transcription factor (WSTF), has been hypothesised to regulate vitamin D receptor trans-repression of the *CYP27B1* gene [3] (Fig. 1).

Several treatment options have been used for hypercalcaemia in IIH and Williams syndrome. Vitamin D supplementation must be stopped immediately in all cases and rehydration performed. Rehydration therapy combined with furosemide is often successful in reducing serum calcium in patients with Williams syndrome; however, some children are unresponsive and require further treatment [65]. Bisphosphonates have been used to treat hypercalcaemia in several cases of Williams syndrome [65, 66]. Serum calcium levels remained normal in these infants and adverse effects were not observed [65, 66]. A number of studies have demonstrated a low calcium diet is effective in treating hypercalcaemia in IIH and Williams syndrome, although this must be closely monitored due to its potential to induce calcium deficiency and rickets [8, 61, 67]. Moreover, low calcium diet may not successfully normalise serum calcium and PTH levels [8]. Often, corticosteroids are used for the treatment of symptomatic hypercalcaemia as they can inhibit enteral calcium absorption and induce *CYP24A1* expression [48]. However, a study of adults with *CYP24A1* mutations showed corticosteroids fail to lower serum calcium levels, normalise PTH levels and decrease urinary calcium excretion in some individuals [52]. Use of corticosteroids should be short to avoid their well-documented side effects [67].

Descriptions of the successful use of calcitonin, which inhibits osteoclastic bone resorption and enhances kidney calcium excretion, have also been described to treat hypercalcaemia, in combination with thiazide diuretics to reduce hypercalciuria [68]. However, repeated administration of calcitonin leads to tachyphylaxis within 48 h, limiting its long-term use [67]. The administration of cellulose phosphate, which binds calcium with high affinity thereby inhibiting intestinal absorption, has also been described to successfully resolve hypercalcaemia in IIH, although this treatment is not available in some countries [67].

### Inborn errors of metabolism

Hypophosphatasia (OMIM #171760) is caused by inactivating mutations in the tissue non-specific isoenzyme of alkaline phosphatase (TNSALP) encoded by the *ALPL* gene [69]. TNSALP hydrolyses pyrophosphate phosphodiesterase to generate inorganic phosphate, which is required for hydroxyapatite formation and skeletal mineralisation [3, 70]. It can be detected biochemically as low serum ALP activity, with mutation analysis of *ALPL* providing confirmation [69].

Hypophosphatasia can present in various forms in neonates and infants and has a prevalence of 1:300,000 in Europe and 1:182,000 in Japan [70]. The most severe form, known as perinatal hypophosphatasia, is characterised by profound skeletal hypo-mineralisation [69]. Infantile hypophosphatasia presents before six months of age [70]. Affected newborns appear healthy, but develop poor feeding, inadequate weight gain, wide fontanels and rachitic deformities that can result in respiratory complications [69]. Hypercalcaemia can occur due to impaired skeletal calcium uptake. In those individuals that survive infancy, persistent rickets, bony craniosynostosis and muscle weakness can occur [69]. Until recently, survival from perinatal hypophosphatasia was rare; however, a recombinant enzyme replacement therapy, asfotase alfa, substantially improves bone mineralisation, respiratory function and survival in perinatal and infantile hypophosphatasia [71]. The severity of hypophosphatasia generally reflects the inheritance pattern of *ALPL* mutations, with autosomal recessive inheritance more common in perinatal and infantile cases [69].

Blue diaper syndrome or Drummond’s syndrome (OMIM #211000) is so called as affected infants have inadequate intestinal tryptophan absorption, leading to excessive metabolism by intestinal bacteria and accumulation of indican, which gives urine a distinctive blue colour [72]. The syndrome was first described in 1964 in two siblings who presented with a blue discoloration of urine, along with hypercalcaemia and nephrocalcinosis [72]. Other symptoms include fever, irritability, constipation, poor appetite and failure to thrive. Some infants present with eye abnormalities including abnormal eye movements, hypoplasia of the optic disc and microcornea [73]. Although the blue discoloration of urine is often the first indication of the syndrome, this symptom has been reported in patients with faecal colonisation with *Pseudomonas aeruginosa* and urinary tract infections [74, 75]. Therefore, a diagnosis requires a thorough clinical evaluation including a detailed patient history and identification of characteristic symptoms to avoid misdiagnosis.

Blue diaper syndrome is extremely rare and therefore estimating incidence is difficult. The inheritance pattern is most likely autosomal recessive. Defects in several proteins that transport amino acids including tryptophan have been proposed, although no mutations in these genes have been identified. Recently, a male infant with blue diaper syndrome with a homozygous frameshift mutation in pro-protein convertase subtilisin/kexin type 1 (*PCSK1*) was described [76]. *PCSK1* encodes prohormone convertase 1/3, a serine endoprotease that is required for the processing of precursors of many hormones including insulin and glucagon-like peptide-1. Mutations in *PCSK1* have previously been associated with obesity and gastrointestinal disorders including malabsorptive diarrhoea [77, 78]. Notably, the boy described in this case did not have hypercalcaemia [76], and further genetic investigation is required to determine whether *PCSK1* mutations are important in other cases of blue diaper syndrome with hypercalcaemia.

Congenital glucose-galactose malabsorption is an autosomal recessive disorder caused by inactivating mutations in the *SLC5A1* gene that encodes the sodium-dependent glucose transporter-1 (SGLT-1), which is predominantly expressed in the intestinal brush-border membrane [79]. Glucose and galactose are produced by enzymatic breakdown of lactose from breast milk at the brush-border membrane, and are then transported by SGLT-1 into the enterocyte, before diffusion across the basolateral membrane via the facilitated carrier GLUT2 [79]. The low intracellular sodium concentration required to maintain these favourable gradients relies on a basolateral Na^+^-K^+^ pump, resulting in net transport of sodium, sugar and water across the epithelium. Consequently, patients with inactivating mutations in SGLT-1 present with severe life-threatening watery diarrhoea dehydration and abdominal distension in the first weeks of life.

The majority of mutations in SGLT-1 are nonsense, frameshift or splice site, resulting in truncated proteins that are not expressed [79]. Missense mutations have been reported, which do not affect protein expression, but impair the transport ability of the protein [79, 80]. Hypercalcaemia occurs in ~20% of cases and can be accompanied by nephrocalcinosis and elevated 1,25(OH)_2_D_3_ [80]. Upregulation of epithelial calcium channels (TRPV6) and 1,25(OH)_2_D_3_ have been hypothesised to contribute to nephrocalcinosis and hypercalcaemia [80]. Restriction of glucose, galactose, sucrose and lactose from the diet rapidly resolves symptoms in most patients.

Another rare autosomal recessive disorder, congenital lactase deficiency, has a similar clinical presentation to glucose-galactose malabsorption [81]. Neonates frequently exhibit hypercalcaemia and nephrocalcinosis. Congenital lactase deficiency is caused by homozygous or compound heterozygous mutations in the gene for lactase (*LCT*). A lactose-free diet usually leads to rapid recovery.

### Additional causes

Subcutaneous fat necrosis of the newborn is another rare cause of hypercalcaemia that normally arises within a few weeks of birth [3, 82]. The condition often occurs following traumatic birth and is characterised by painful, firm nodules over the body that are hypothesised to form following cold- or stress-induced injury to immature fat, resulting in necrosis and granulomatous infiltrate [82]. Subcutaneous fat necrosis can occur in newborns with hypoxic-ischemic encephalopathy treated with moderate therapeutic hypothermia [83]. Although subcutaneous fat necrosis usually self-resolves, the associated hypercalcaemia, caused by a combination of calcium release from necrotic tissue and excess calcitriol and prostaglandin E activity from the granulomatous reaction, can be fatal [3]. Hypercalcaemia associated with subcutaneous fat necrosis has been shown to respond to furosemide and glucocorticoid treatment, although an increased incidence of nephrocalcinosis may occur in these infants. Bisphosphonate treatment successfully resolved serum calcium levels and had a reduced risk of recurrent hypercalcaemia [84].

Hypercalcaemia has been reported in neonates with the kidney tubular disorder Bartter’s syndrome. The affected infants had mutations in the sodium-potassium-chloride co-transporter NKCC2 (encoded by *SLC12A1*) and the potassium channel ROMK (encoded by *KCNJ1*) [85, 86]. Patients with this form of Bartter’s, occasionally known as hyperprostaglandin E syndrome, have increased release of prostaglandin E_2_ (PGE_2_) in the plasma and urine. The basis of hypercalcaemia in these patients is uncertain, although elevated PGE_2_ may contribute as studies have shown it can stimulate bone resorption, increase PTH secretion and upregulate hydroxylation of 25-hydroxyvitamin D [86].

Hypercalciuria and/or hypercalcaemia can occur in children with the intrauterine growth retardation, metaphyseal dysplasia, adrenal hypoplasia congenita and genital anomalies IMAGe syndrome (OMIM #614732). Heterozygous mutations in the cyclin-dependent kinase inhibitor 1C (*CDKN1C*) gene have been identified in some cases, and inheritance may involve maternal imprinting [87]. As only a few cases have been reported, the cause of hypercalcaemia is unclear.

Hypercalcaemia may occur in association with a number of endocrine disorders including hyperthyroidism, hypothyroidism and adrenal insufficiency. Neonatal hyperthyroidism is usually associated with maternal Graves’ disease but can occur in rare endocrine disorders including autosomal dominant non-autoimmune hyperthyroidism due to activating mutations in the TSH receptor gene, McCune-Albright syndrome due to activating mutations in the *GNAS* gene, encoding the G-protein alpha-s and mutations in the thyroid receptor beta gene. Mild hypercalcaemia has been reported in infants with congenital hypothyroidism. Treatment of these infants with levothyroxine increases circulating 1,25(OH)_2_D_3_ and hypercalciuria, contributing to hypercalcaemia [88]. In patients with adrenal insufficiency, hypercalcaemia may be related to reduced calcium reabsorption at the proximal tubule due to hypovolemia and consequent reduced glomerular filtration rate and/or due to increased 1α-hydroxylase activity [89]. Rare cases of PTHrP-secreting tumors have been reported in neonates. PTHrP acts on the PTH1R to cause hypercalcaemia. A number of other causes of hypercalcaemia have been reported in neonates and infants including medications (such as thiazides and lithium) and extremely rare disorders, which have been well described in a review from 2005 [90].

#### Key Summary Points

- Serum calcium levels are higher in neonates than older children and adults. Therefore age-appropriate normal ranges should be used.
- Transient hypercalcaemia may occur shortly after birth, caused by maternal hypocalcaemia.
- Accurate identification of the cause of hypercalcaemia may require combined assessment of the family history; biochemical parameters (serum calcium, phosphate, creatinine and albumin, PTH, vitamin D metabolites and urinary calcium, phosphate and creatinine); evaluation of physical features (to identify dysmorphology); and genetic analyses.
- As next-generation sequencing continues to improve, it is likely that further hypercalcaemia-associated genes will be identified. This may yield new treatment avenues and continue to improve neonatal care.

#### Multiple choice questions (answers are provided following the reference list)

1. Neonatal severe hyperparathyroidism (NSHPT) and familial hypocalciuric hypercalcaemia (FHH) are:
  - Both caused by inactivating mutations in the CaSR gene only.
  - NSHPT is caused by inactivating mutations in the CaSR only; FHH is caused by inactivating mutations in the CaSR, *Gα11* and *AP2σ* genes.
  - NSHPT is caused by inactivating mutations in the CaSR, *Gα11* and *AP2σ* genes; FHH is caused by inactivating mutations in the CaSR gene only.
  - Both are caused by inactivating mutations in the CaSR, *Gα11* and *AP2σ* genes*.*
2. Jansen’s metaphyseal chondrodysplasia is characterised by:
  - Short-limbed short stature, deformed, under-mineralised bones, chronic hypercalcaemia and hypophosphaturia with elevated serum PTH levels and elevated serum markers of bone turnover.
  - Short-limbed short stature, deformed, under-mineralised bones, chronic hypercalcaemia and hypophosphaturia with normal serum PTH levels and elevated serum markers of bone turnover.
  - Short-limbed short stature, deformed, under-mineralised bones, transient hypercalcaemia and hyperphosphaturia with normal serum PTH levels and elevated serum markers of bone turnover.
  - Short-limbed short stature, deformed, under-mineralised bones, chronic hypercalcaemia and hyperphosphaturia with normal serum PTH levels and elevated serum markers of bone turnover.
3. Idiopathic infantile hypercalcaemia (IIH) is caused by mutations in:
  - The *CYP27B1* gene, encoding 1α-hydroxylase, which converts 25-hydroxyvitamin D3 to active 1,25(OH)_2_D_3_.
  - The *CYP24A1* gene, encoding 24-hydroxylase, which inactivates 1,25(OH)_2_D_3_.
  - The *CYP2R1* gene, encoding 25-hydroxylase, which converts vitamin D to 25-hydroxyvitamin D3.
  - The *CYP24A1* and *CYP27B1* genes.
  - The *CYP2R1*, *CYP24A1* and *CYP27B1* genes.
4. In Williams syndrome the reported frequency of hypercalcaemia is:
  - >50%, is often symptomatic, may resolve within the first few years, but may recur during puberty.
  - <50%, is often symptomatic, may resolve within the first few years, but may recur during puberty.
  - <50%, is often asymptomatic, and always resolves within the first few years, with no recurrence.
  - >50%, is often asymptomatic, and always resolves within the first few years, with no recurrence.
5. Which of these causes of hypercalcaemia is characterised by poor feeding, rachitic deformities that can result in respiratory complications, persistent rickets, bony craniosynostosis and is associated with mutations in the *ALPL* gene:
  - Jansen’s metaphyseal chondrodysplasia
  - William’s syndrome
  - Hypophosphatasia
  - Blue diaper syndrome
  - Subcutaneous fat necrosis of the newborn
